# Genome engineering of stem cell organoids for disease modeling

**DOI:** 10.1007/s13238-016-0368-0

**Published:** 2017-01-19

**Authors:** Yingmin Sun, Qiurong Ding

**Affiliations:** 1 CAS Key Laboratory of Nutrition and Metabolism, Institute for Nutritional Sciences, Shanghai Institutes for Biological Sciences, Chinese Academy of Sciences, Shanghai, 200031 China; 2 University of Chinese Academy of Sciences, Shanghai, 200031 China

**Keywords:** pluripotent/adult stem cell, tissue organoid, genome editing, precision medicine

## Abstract

Precision medicine emerges as a new approach that takes into account individual variability. Successful realization of precision medicine requires disease models that are able to incorporate personalized disease information and recapitulate disease development processes at the molecular, cellular and organ levels. With recent development in stem cell field, a variety of tissue organoids can be derived from patient specific pluripotent stem cells and adult stem cells. In combination with the state-of-the-art genome editing tools, organoids can be further engineered to mimic disease-relevant genetic and epigenetic status of a patient. This has therefore enabled a rapid expansion of sophisticated *in vitro* disease models, offering a unique system for fundamental and biomedical research as well as the development of personalized medicine. Here we summarize some of the latest advances and future perspectives in engineering stem cell organoids for human disease modeling.

## Introduction

The concept of precision medicine has been “hot” again in the public spotlight in recent years. The fundamental idea behind precision medicine is to integrate information from all aspects to create diagnostic and therapeutic strategies that are precisely tailored to each patient’s requirement (Collins and Varmus, 2015). Recent advances in biotechnologies have led to an explosion of disease relevant molecular information, providing enormous opportunities to obtain the pathophysiological insights to develop personalized treatment. However, challenges exist that a model system is urgently needed for interpreting the information. An ideal system would have the following several features: 1) could recapitulate human body functions and disease development processes at different levels of molecular, cellular, tissue or organ; 2) could incorporate personalized disease information (e.g. genetic background, nutritional status, environmental disturbance, etc.); and 3) could be cultured in large scales for disease study and high-throughput drug screenings.

The discovery that mature cells can be reprogrammed to become pluripotent by Drs. John Gurdon and Shinya Yamanaka has opened the door that pluripotent stem cells from patients can be generated for disease modeling (Takahashi et al., 2007; Yu et al., 2007). Human pluripotent stem cells (hPSCs) as well as adult stem cells (ASCs) derived from tissues offer a unique *in vitro* cellular model system with several advantages. First, they have a human genome and thus are the most appropriate for studying human disease-relevant genetic variations. Second, under appropriate culture conditions, the cells can be directed to differentiate into a variety of terminal cell types. And the *in vitro* differentiation process often recapitulates aspects of normal development, providing the opportunity to study developmental and degenerative processes. Third, they can be maintained in culture for a large number of passages while keeping the genome integrity, thus are able to generate cellular materials in a large-scale for compound screenings.

In recent years, stem cells have been revealed to possess intrinsic abilities to generate complex structures *in vitro* through self-organization (Eiraku et al., 2008; Gaspard et al., 2008; Sato et al., 2009). When cultured in a 3D space and in the presence of suitable exogenous factors, stem cells form structures that mimic the architecture of a certain organ *in vitro*—these 3D complex structures are thus termed “organoids”. Compared to 2D monocultures of a single cell type, organoids have the potential to recapitulate spatial organization of heterogeneous tissue-specific cells, cell-cell and cell-matrix interactions. Moreover, the emergence of genome editing technologies over the past a few years has made it feasible to efficiently engineer disease relevant genetic variations into stem cells (Hsu et al., 2014; Wang and Qi, 2016). Therefore, different organoids can be generated carrying patient specific disease-causing genetic and epigenetic background. Altogether, this is offering a unique model system for studying disease-relevant information and development of precision medicine. In this review, we discuss how human stem cells are self-organized to form different kinds of organoids, and how these tissue organoids, in combination with genome editing tools, can leverage our understanding of human disease development and will facilitate the realization of precision medicine. We will also highlight some of the existing limitations or challenges as well as future directions.

## Organoid Formation

In general, organoids can be generated either from ASCs derived from tissues, or from PSCs, including patient-derived induced pluripotent stem cells (iPSCs). Stem cells are often placed within a hydrogel (e.g. Matrigel) in a 3D culture system, with supplement of suitable exogenous factors to mimic biochemical and physical cues of tissue development. Cells then exhibit some intrinsic ability to assemble into complex structures termed as organoids. These organoids are constituted of more than one cell type, often resemble architecture of a certain organ and exhibit some function specific to that organ, and this *in vitro* formation process is termed as “self-organization”.

### Organoids derived from ASCs

The development of the intestinal organoid culture system in 2009 was a major technological advance in this field (Sato et al., 2009). In this study, single Lgr5^+^ stem cells were isolated from mouse intestinal epithelium and cultured in a simple 3D culture system in which Matrigel was used as extra cellular matrix. A few key growth factors, such as WNT, Noggin, R-spondin and EGF, were supplemented mostly to maintain stem cell populations. Remarkably, these stem cells were found later to be able to form continuously expanding crypt-villus organoids that present near-physiological epithelium architecture reminiscent of normal gut. A similar culture system was subsequently adapted for generation of human intestinal organoids (Dekkers et al., 2013). Moreover, Lgr5^+^ stem cells derived from other organs were also found later to be able to self-organize to form different organoids, including stomach, colon and liver (Barker et al., 2010; Huch et al., 2013a, b; Sato et al., 2011; Stange et al., 2013). In summary, organoids that have been successfully generated with ASCs from primary murine or human tissues include intestine (mouse/human) (Dekkers et al., 2013; Sato et al., 2009; Yin et al., 2014), stomach (mouse/human) (Barker et al., 2010; Bartfeld et al., 2015; Schlaermann et al., 2016; Stange et al., 2013), oesophagus (mouse/human) (DeWard et al., 2014; Sato et al., 2011), colon (mouse/human) (Jung et al., 2011; Sato et al., 2011), liver (mouse) (Huch et al., 2013b; Huch et al., 2015), pancreas (mouse/human) (Boj et al., 2015; Huch et al., 2013a), lingual (mouse) (Hisha et al., 2013), lung (mouse) (Lee et al., 2014; Mondrinos et al., 2006; Mondrinos et al., 2014), taste bud (mouse) (Aihara et al., 2015; Ren et al., 2014), salivary gland (mouse) (Nanduri et al., 2014) and prostate (mouse/human) (Chua et al., 2014; Gao et al., 2014; Karthaus et al., 2014) (Table 1).

**Table 1:** Summary of organoids derived from ASCs

Tissue	Cell source	Main growth factors used #	3D structure or organoid	Ref.
Intestine	Lgr5^+^ stem cells from small intestinal crypts	EGF, Noggin, R-spondin1, Wnt3a	Crypt-villus branching organoids	Dekkers et al., 2013; Sato et al., 2009; Yin et al., 2014
Stomach	Lgr5^+^ stem cells from pyloric glands	EGF, Noggin, R-Spondin1, Wnt3a, FGF10	Gastric organoid resembling pyloric gastric unit	Barker et al., 2010; Bartfeld et al., 2015; Schlaermann et al., 2016; Stange et al., 2013
Oesophagus	Biopsy sample from Barrett’s epithelium	EGF, Noggin, R-Spondin1, Wnt3a, FGF10	Spherical or budding oesophagus organoid	DeWard et al., 2014; Sato et al., 2011
Colon	Epithelial colonic stem cells	EGF, Noggin, R-Spondin1, Wnt3a, nicotinamide	Spherical or budding colonic organoid	Jung et al., 2011; Sato et al., 2011
Liver	Lgr5^+^ liver stem cells or EpCAM^+^ bile duct cells	EGF, Noggin, R-Spondin1, Wnt3a, FGF10, HGF, nicotinamide	Spherical or cystic hepatic organoid	Huch et al., 2013b; Huch et al., 2015
Pancreas	Isolated pancreatic duct fragments	EGF, Noggin, R-Spondin1, Wnt3a, FGF10, nicotinamide	Budding cyst‐like or branching pancreatic organoid	Boj et al., 2015; Huch et al., 2013a
Lingual	Bmi1-positive stem cells from lingual epithelium	EGF, Noggin, R-Spondin1	Spherical or budding lingual organoid	Hisha et al., 2013
Lung	Bronchioalveolar stem cells	Co-culture with primary lung/liver endothelial cells	Spherical bronchioalveolar structures	Lee et al., 2014; Mondrinos et al., 2006; Mondrinos et al., 2014
Taste bud	Lgr5^+^ or Lgr6^+^ taste stem cells	EGF, Noggin, R-Spondin1, Jagged-1	Spherical or budding taste organoid	Aihara et al., 2015; Ren et al., 2014
Salivary gland	Salivary gland stem cells	EGF, FGF-2, insulin, dexamethasone	Ductal or lobular minigland organoid	Nanduri et al., 2014
Prostate	Luminal epithelial progenitor from prostate	EGF, Noggin, R-Spondin1, TGF-β/Alk inhibitor	Spherical prostate organoid	Chua et al., 2014; Gao et al., 2014; Karthaus et al., 2014

### Organoids derived from PSCs

The use of PSCs to generate organoids circumvents the limitation of availability of human primary tissues where ASCs are derived from. Protocols are often established following the knowledge of developmental biology with manipulation of key growth factors or signaling pathways involved in germ layer and subsequent lineage specification. The first organoid generated from mouse and human PSCs are cortical tissues that displayed nicely the patterned structures mimicking the early aspect of cortical development (Eiraku et al., 2008; Gaspard et al., 2008). These studies have demonstrated the intrinsic ability of corticogenesis of stem cells when cultured in three-dimensional system with appropriate exogenous signals. From there, a variety of organoids of different tissues have been generated. The culture system in general is similar with the one used for organogenesis from ASCs, in which the proper 3D scaffold and biochemical factors are provided to induce the differentiation of PSCs down specific lineages, and differentiated cells will self-organize to form tissue-specific organoids. Current organoids that have been reported to be generated from mouse or human PSCs include stomach (mouse/human) (McCracken et al., 2014; Noguchi et al., 2015), intestine (mouse/human) (McCracken et al., 2011; Ootani et al., 2009; Spence et al., 2011; Watson et al., 2014; Workman et al., 2016), liver (human) (Ogawa et al., 2015; Sampaziotis et al., 2015; Takebe et al., 2013; Takebe et al., 2014), lung (human) (Dye et al., 2015), retina (mouse) (Eiraku et al., 2011), inner ear (mouse) (Koehler et al., 2013; Koehler and Hashino, 2014), brain (mouse/human) (Eiraku et al., 2008; Gaspard et al., 2008; Lancaster et al., 2013; Lancaster and Knoblich, 2014; Mariani et al., 2012; Muguruma et al., 2010, 2015; Qian et al., 2016), pituitary gland (mouse/human) (Ozone et al., 2016; Suga et al., 2011) and kidney (human) (Takasato et al., 2015) (Table 2).

**Table 2:** Summary of organoids derived from PSCs

Tissue	Main growth factors used #	3D structure or organoid	Ref.
Intestine	ActivinA, Wnt3a, FGF4, EGF, Noggin, R-spondin1	Crypt-villus branching organoids	McCracken et al., 2011; Ootani et al., 2009; Spence et al., 2011; Watson et al., 2014; Workman et al., 2016
Stomach	Activin A, Wnt3a, CHIR99021, FGF4, Noggin, retinoic acid	Spherical or budding gastric organoid	McCracken et al., 2014; Noguchi et al., 2015
Liver	Activin A, BMP4, FGF2, HGF(H^*^), Oncostatin M (H^*^), FGF10 (C^*^), retinoic acid (C^*^), EGF (C^*^)	Spherical or cystic hepatic organoid	Ogawa et al., 2015; Sampaziotis et al., 2015; Takebe et al., 2013; Takebe et al., 2014
Lung	Activin A, Wnt3a, FGF4, Noggin, SB431542, SU5402, Sant-2, SAG, SHH	Spherical lung organoid	Dye et al., 2015
Retina	Wnt3a, Nodal, DAPT, retinoic acid	Spherical retina organoids	Eiraku et al., 2011
Inner ear	BMP4, SB431542, FGF2, LDN193189	Spherical or budding inner ear organoid	Koehler et al., 2013; Koehler et al., 2014)
Brain	Forebrain: dorsomorphine, A83-01, Wnt3a, CHIR99021, SB431542, BDNF, GDNF, TGFβ, c-AMP; Midbrain: LDN193189, SB431542, SHH, purmorphamine, FGF-8, CHIR99021, BDNF, GDNF, TGFβ, c-AMP; Hypothalamus: LDN193189, SB431542, 1-Thioglycerol, Wnt3a, SHH, purmorphamine, FGF-2	Spherical or budding brain organoid	Eiraku et al., 2008; Gaspard et al., 2008; Lancaster et al., 2013; Lancaster et al., 2014; Mariani et al., 2012; Muguruma et al., 2010, 2015; Qian et al., 2016
Pituitary gland	BMP4, dorsomorphin, SAG, Wnt4, Wnt5, FGF8, DAPT, Nodal, IWP2	Spherical Rathke’s-pouch-like organoid	Ozone et al., 2016; Suga et al., 2011
Kidney	FGF9 (or FGF2), CHIR99021, retinoic acid	Spherical or budding kidney organoid	Takasato et al., 2015

#: Different combinations of growth factors may be applied in different labs and between human and mouse organoids

H*: Growth factors specifically used for hepatocyte differentiation

C*: Growth factors specifically used for cholangiocytes differentiation

BDNF: brain derived neurotrophic factor; BMP: bone morphogenetic protein; CHIR99021: GSK-3β inhibitor; dorsomorphin, A83-01, SB431542, LDN193189: SMAD inhibitors; DAPT: notch inhibitor; EGF: epidermal growth factor; FGF: fibroblast growth factor; GDNF: glial cell-derived neurotrophic factor; HGF: hepatocyte growth factor; IWP2: Wnt inhibitor; purmorphamine: SHH agonist; SAG: smoothened agonist, hedgehog agonist; Sant-2: hedgehog inhibitor; SHH: sonic hedgehog; SU5402: FGF receptor inhibitor

### Construction of vascular and nervous systems

While a wide variety of organoids have been generated, there are still huge gaps between *in vitro* generated organoids and *in vivo* tissues. For example, organs *in vivo* often consist of hierarchically branched vascular networks. Almost all cells within an organ are surrounded by a few hundred microns of a capillary to permit sufficient nutrient and oxygen supply. Also organs *in vivo* have integrated peripheral nervous system that connects with the central nervous system. The nervous system in general serves as a communication relay, monitoring and coordinating internal organ function and responds to changes in the external environment. Thus, the construction of integrated vascular network and peripheral nervous system in organoids are important considerations in developing functional *in vitro* tissues.

There have been several approaches established for functional vascularization of generated organoids. One approach is to seed endothelial cells within the culture system that allows formation of blood vessels (Takebe et al., 2013; Takebe et al., 2014). An example comes from the generation of vascularized liver bud, in which hepatic endoderm cells derived from hPSCs were co-cultured with human umbilical vein endothelial cells (HUVECs) and human mesenchymal stem cells (MSCs) to form “liver bud”. Upon transplantation into mice, these liver buds connected with the host vasculature and formed functional vascular networks similar in density and morphology to those of human adult livers. The other approach involves synthetic scaffolds, which are used to create micro-engineered 3D structures. Bio-printing methods can then be used for seeding the channels with multiple types of vascular cells (Visconti et al., 2010; Yin et al., 2016). For reconstruction of integrated nervous system in organoids, the first successful work was recently published that presented engineered hPSC-derived intestinal tissues with a functional enteric nervous system (Workman et al., 2016). The strategy taken was similar with seeding endothelial cells for vascularization. hPSC-derived neural crest cells (NCCs) and human intestinal organoids were mixed together through low-speed centrifugation in 3D growth conditions. Upon transplantation *in vivo,* these organoids formed neuroglial structures similar to a myenteric and submucosal plexus, and moreover, displayed functional interstitial cells of Cajal as well as electromechanical coupling that regulated waves of propagating contraction.

Other than lack of complete vascular and nervous systems, there are still key components lacking in current organoids. For instance, stromal components including immune cells that have important disease implications are missing, which has largely limited the applications in modeling inflammatory responses (e.g. to infection or drugs). Additional challenges exist that the underlying mechanisms that how stem cells self-organize remains obscure. It is therefore often difficult to control the cell type, cell organization, cell-cell and cell-matrix interactions. Recent advances in biomaterials, micro/nanotechnology and bioengineering approaches offer some promising solutions (Yin et al., 2016). Biomimetic systems can be engineered to incorporate extrinsic biochemical and biophysical signals to modulate the stem cell niche, to control cell-cell and cell-matrix interaction, oxygen distribution, local pH levels and nutrient transport within organoids (Yin et al., 2016).

## Genome Engineering

While organoids can be generated from stem cells isolated directly from patient’s tissues or from patient iPSCs for disease modeling, appropriate control is required to establish disease-relevant phenotypes for mechanism study and compound screenings. Availability of strict control groups is becoming even more important in organoid systems, in which huge systematic variations exist due to complicated processes including derivation and differentiation of stem cells as well as diverse engineering tools used for organoid formation. The most rigorous possible control would be a cell line that differ only with respect to disease mutations compared to the original cell line, i.e., otherwise isogenic cell lines. Such a strategy would require the ability to efficiently introduce specific genetic alterations into the genomes of hPSCs at will.

Fortunately, the discovery of different targeted nucleases in recent years, especially the clustered regularly interspaced short palindromic repeats (CRISPR)/CRISPR-associated (Cas) system, has enabled researchers to accurately manipulate genomic sequences in different cells and model organisms. Within a couple of years, genome-editing technology has been rapidly developed for a variety of research and translational applications, making it an extremely powerful tool that can facilitate disease modeling by recapitulating the genetic defects.

Although with different working modules, most of the genome-editing tools share the same editing methodology by introducing double stranded-breaks (DSBs) into a desired genomic site. Cells react to DSBs by initiating DNA repair processes, either through non-homology end joining (NHEJ) or homology directed recombination (HDR). DNA repaired by NHEJ is error-prone, which can introduce small insertions or deletions (indels) into a genomic locus—causing frameshift thus knocking out the gene in coding region, or destroying a DNA regulation element in non-coding region; whereas DNA repaired by HDR can be used to introduce precise single nucleotide alterations or insert a whole gene into a specific genomic locus by providing an exogenous DNA template. When multiple DSBs have been made simultaneously into the cells (so called “multiplex”), chromatin structure changes, such as truncation, or even translocation or duplication, can also be made, albeit at a relatively lower rate (Hsu et al., 2014; Wang and Qi, 2016).

**Figure 1:**
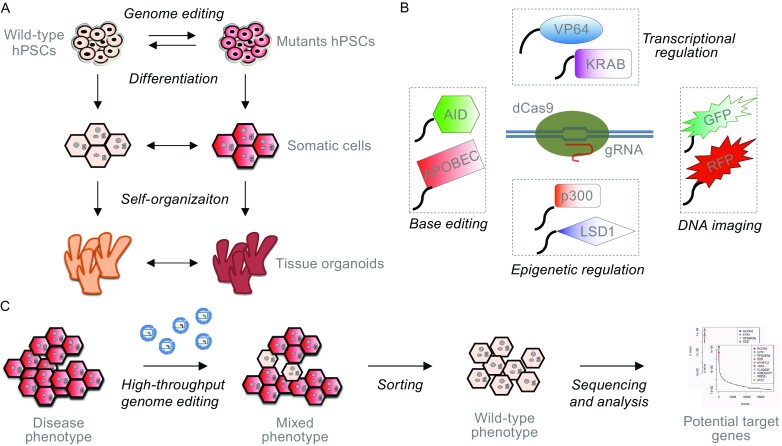
Applications of genome engineering in disease modeling. With genome editing tools, stem cell lines from patients can be efficiently “cured” to correct the disease relevant genetic variants; or wild-type stem cells can also be introduced with genetic variants to create “patient” cell lines for disease modeling (A). Catalytically dead nucleases, such as deactivated Cas9 (dCas9), can be fused to different functional effectors and carry out molecular functions other than genome editing (B). High-throughput genetic screenings can be developed using genome editing tools to illuminate genes or signaling pathways involved in disease development (C)

With genome editing tools, stem cell lines from patients can be efficiently “cured” to correct the disease relevant genetic variants to establish isogenic control groups (Fig. 1A). In the case when patients carrying certain genetic mutations are not accessible, wild-type stem cells can also be introduced with genetic variants to create “patient” cell lines (Fig. 1A). Indeed, with this strategy a variety of genetic variants, either in the same gene or in multiple genes, can be introduced into a single cell line so that they are on the same genetic and epigenetic background (Ding et al., 2013). This allows for direct comparison of the effects of the different genetic variants, setting up a panel of disease models for study of mutation-specific disease phenotypes and for screening of tailored disease treatments (Musunuru, 2013).

Not only genome editing tools can be used for engineering DNA sequences, taking advantage of their ability to target a specific genomic region, catalytically dead nucleases, such as deactivated Cas9 (dCas9), can be fused to functional effectors and carry out functions other than genome editing (Wang and Qi, 2016) (Fig. 1B). For example, dCas9 has being fused with transcriptional activators or repressors to regulate gene expression at the transcriptional levels (Cheng et al., 2013; Gilbert et al., 2013; Perez-Pinera et al., 2013a; Pinera et al., 2013b; Qi et al., 2013); with epigenetic regulators (e.g. acetyltransferase and histone demethylase) to manipulate the epi-status of a genomic region (Amabile et al., 2016; Hilton et al., 2015; Kearns et al., 2015; Thakore et al., 2015); with fluorescent proteins (e.g. green fluorescent protein, GFP) for imaging of specific DNA sequences (Chen et al., 2013; Ma et al., 2016a, b; Nelles et al., 2016; Schmidt et al., 2016); and more recently with cytidine deaminase enzymes to introduce the C->T (or G->A) transitions, offering a more efficient approach for base editing than HDR (Komor et al., 2016; Ma et al., 2016a, b; Nishida et al., 2016).

Besides making specific genetic or epigenetic alterations to cells or model organisms, genome-editing tools, especially CRISPR/Cas9, have also been developed for high-throughput genetic screenings (Fig. 1C). A variety of gRNA libraries are currently available through Addgene (www.addgene.org) that allows to screen tens of thousands of genes genome-wide or a specialized group of genes (e.g. kinases, transcription factors) for gene targets that are responsible for a biological phenotype of interest. For instance, screens have been performed to dissect genes or signaling pathways involved in the development of tumor drug resistance (Koike-Yusa et al., 2014; Konermann et al., 2015; Gilbert et al., 2014; Shalem et al., 2014; Wang et al., 2014; Zhou et al., 2014), transformation and metastasis of cancer cells (Chen et al., 2015), pyroptotic cell death (Parnas et al., 2015; Shi et al., 2015) or mitochondrial deficiency (Jain et al., 2016). Guide RNA libraries can also be customized to target a specific non-coding genomic region in order to dissect potential DNA regulating elements for a certain cellular phenotype in a high-throughput manner (Canver et al., 2015; Sanjana et al., 2016). Moreover, a paired-guided RNA CRISPR/Cas9 library has recently been generated to screen for lincRNAs by performing multiplex targeting to delete the entire lincRNA sequences (Zhu et al., 2016). All these applications can greatly facilitate the manipulation of genetic and epigenetic status in organoids for disease modeling as well as obtaining the pathophysiological insights for disease development and treatment.

## Disease Modeling

Disease modeling with stem cells, especially with patient derived iPSCs, has been applied to a variety of human diseases (Musunuru, 2013). Although current stem cells-generated disease models mostly rely on 2D monocultures of a single cell type, these cellular models have been proven to be valuable in modeling human diseases with high physiological relevance, and meantime offering abundant cellular materials for high-throughput compound and genetic screenings. One of the best examples came from studies of human premature aging. One study focused on Hutchinson-Gilford progeria syndrome (HGPS) disease, which is caused by mutation in the *LMNA* gene. In this study, patient iPSC and isogenic control iPSC lines were generated and differentiated into vascular smooth muscle cells and fibroblasts for disease modeling (Liu et al., 2011). Furthermore, by setting up a high-throughput siRNA screen with these cells, suppressed NRF2 antioxidant activity has been successfully identified as a driver mechanism in HGPS (Kubben et al., 2016). Instead, a different study also focused on human premature aging used a stem cell model generated by knocking out exons of the *WRN* gene in wild-type embryonic stem cell lines and subsequently differentiated to MSCs to mimic the Werner syndrome (WS) (Zhang et al., 2015). Studies with this cell model revealed a significant role for *WRN* in maintaining heterochromatin stability. A chemical screen was later performed on these *WRN*-deficient MSCs, and interestingly, Vitamin C was found to be able to rescue many of the aging features, suggesting it be a potential treatment to alleviate Werner syndrome (Li et al., 2016).

**Figure 2:**
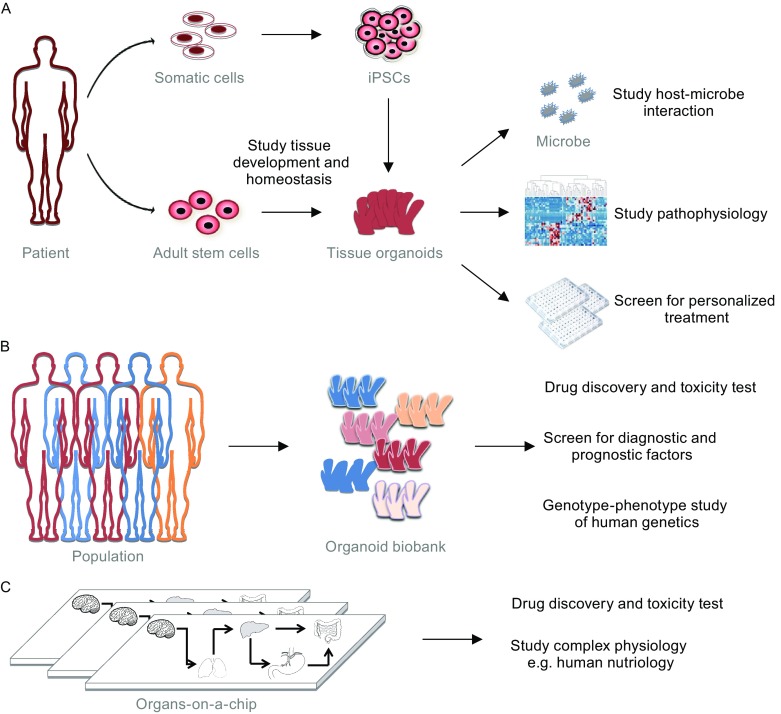
Applications of organoid technology in basic and translational research. Tissue organoids can be derived from patient iPSCs or ASCs. The *in vitro* development of organoids offers a cellular system for studying the contribution of various signaling pathways in human tissue development and homeostasis. Established organoids can be used as a model system to study infectious diseases or tissue specific responses to toxins. Organoids with patient-specific disease information can also be generated for pathophysiology study, and can be expanded in a large scale for discovery of personalized treatment through high-throughput screenings (A). Biobanks with organoids generated from human populations are being established, which will provide high valuable resources that can be used to carry out preclinical efficacy and toxicity test of candidate drugs, screen for diagnostic and prognostic factors, and delineate genotype-phenotype causality in conjunction with current genetic studies (e.g. GWAS) (B). Complemented with advances in bioengineering approaches, “organs-on-a-chip” can be built up containing multiple tissues that can offer an efficient system for drug discovery and study of more complex physiological processes, such as human nutriology (C)

With the recent development of organoid culture systems, three-dimensional organoids offer new opportunities for biomedical research in that diseases can be modeled at the organ or tissue level. One of the most developed model system with 3D organoids is from the intestine, representing beautiful multiple approaches by which organoid disease models can be generated and used to study diseases (Fig. 2).

### Infectious diseases

As mucin-rich barriers are established inside 3D organoids from intestine, intestinal organoids that derived either from crypt or PSCs offered a unique cellular system to study pathophysiology of bacterial-epithelia interactions (Forbester et al., 2015; Leslie et al., 2015; Wang et al., 2015; Wilson et al., 2015; Zhang et al., 2014). Upon the invasiveness of bacterial (e.g. strains of *Salmonella* and *Clostridium*), morphologic changes and disruption of epithelial tight junctions were observed in infected organoids, presenting one nice experimental system to study host-bacterial interactions. Similar studies have also been performed to study *Helicobacter pylori* infection using gastric organoids, demonstrating the potential for wider studies of the human microbiome using 3D organoid models of epithelial tissues (Bartfeld et al., 2015; Wroblewski et al., 2015).

### Hereditary diseases

There have been several studies in which intestinal organoids carrying specific disease-relevant mutations were generated for pathophysiological study and for screening of new pharmaceutical interventions. For example, a simple and robust assay that allows the diagnosis, functional study and identification of personalized medicine was developed using intestinal organoids derived from people who suffer from cystic fibrosis (Dekkers et al., 2013). In another study focusing on multiple intestinal atresia (MIA)—a rare cause of bowel obstruction, mutations in tetratricopeptide repeat domain-7A (TTC7A) were identified from patients using linkage analysis and whole-exome sequencing (Bigorgne et al., 2014). Further studies using intestinal organoids derived from MIA patients revealed an inversion of apicobasal polarity of the epithelial cells due to aberrantly increased Rho kinase activity, suggesting a potential treatment with pharmacological inhibition of Rho kinase. Another recent study focused on Hirschsprung’s disease with an impaired neural crest development being the underlying cause (Workman et al., 2016). Study was carried out using the most advanced intestinal organoids that have incorporated a functional enteric nervous system. With these organoids, the molecular basis of pathophysiology of Hirschsprung’s disease caused by a mutation in the *PHOX2B* gene was carefully investigated.

### Cancer

Organoids can also be adapted to develop *in vitro* models of several types of malignancies from intestine/colon, stomach, pancreas, prostate, brain and lung (Schweiger and Jensen, 2016). Cancer organoids can be directly generated from patients’ primary cells, offering a model system that can incorporate patient specific disease information. With such a system, screens can be performed for treatments with high efficacy and minimal side effect (van de Wetering et al., 2015). Organoids can also be used to study the role and requirement of known driven mutations in cancer development (Schweiger and Jensen, 2016). For instance, in one study the most common mutations in colorectal cancer were introduced to wild-type human intestinal organoids with different combinations (Drost et al., 2015). Results revealed that organoids carrying mutants in all 4 genes (*APC*, *TP53*, *KRAS* and *SMAD4)* can grow self-sufficiently and form solid tumors with features of invasive carcinoma upon subcutaneous engrafting, whereas combined loss of *APC* and *P53* was sufficient to induce extensive aneuploidy. A similar study compared the metastasis ability of intestinal organoids carrying mutations in all 5 genes (*APC*, *TP53*, *KRAS*, *SMAD4* and *PI3KCA)* with another engineered organoids derived from patient-derived chromosome-instable adenomas (Matano et al., 2015). Results indicated that although these five mutations are sufficient for tumor development, additional molecular lesions are required for invasive behavior. Similar approach can be extremely useful for dissecting the causality of a specific mutation and/or combinations of mutations identified in individual patients as well as in large cancer genomic studies.

### Neurological diseases

Besides disease models generated with intestine organoids, organoids derived from other tissues have also being used for modeling diseases, such as liver, stomach and pancreas etc. (Drost et al., 2015). In particular, brain organoids can be generated that can be used to model various neurodevelopmental or neurodegenerative disorders that have been difficult or impossible to model in animals. For example, brain organoids derived from patient iPSCs have been used to study the developmental pathogenesis of microcephaly caused by a mutation in CDK5RAP2 (Lancaster et al., 2013). With further development, it will not be surprising that cerebral organoids can also be applied to model and study disorders such as autism (Mariani et al., 2015), virus exposure (Qian et al., 2016) and even adult-onset disorders like neurodegenerative diseases in the future. It is worth mentioning that unlike cultured primary neurons, PSCs can be maintained for a large number of passages, thus these diseased brain organoids can be expanded for high-throughput screenings for compounds for disease treatment, offering a model system with huge potential in drug discovery to brain diseases.

It is clear that the generation of various 3D organoids provides unprecedently a sophisticated model system that has the potential to model a wide variety of human diseases; still limitations exist at least with current systems. Compared with monolayer model systems, which can be readily standardized, 3D organoids generation combines differentiation of stem cells, aggregation of multi-types of cells and diverse bioengineering approaches, making it extremely difficult for standardization and hard for groups to compare results between systems. Another challenge comes from the maturation of organoids *in vitro*. Although 3D organoids displayed in general more matured cellular phenotypes compared to monolayer-cultured cells, most of organoids cannot approach fully maturation until transplanted *in vivo*. Furthermore, microenvironment and hormone levels, the local pH levels, the nutrition status as well as the microbiome of the human body cannot be fully reproduced in an *in vitro* organoid. Thus it is critically important to have thorough characterization of the *in vitro* organoid system with regard to the extent of recapitulation of *in vivo* development before it is used for biomedical research and therapeutics. Besides, incorporation of functional vascular and nervous systems, as well as stromal components for inflammatory studies in organoids still await more development.

## Outlook

The common failures to translate promising preclinical drug candidates into clinical success and the endeavors to realize precision medicine highlight the urgent call for a model system with easy accessibility, high physiological relevance and ability to incorporate patient-specific disease background. Organoids derived from stem cells have proven to be such a system that has the potential to recapitulate crucial aspects of development, homeostasis and disease of human body and to inherit patient-specific disease information as well as has the capacity to expand indefinitely.

With these advantages, organoids technology provides a unique model system for a wide range of applications in both basic research and translational studies. The *in vitro* development of organoids itself offers a cellular system for studying the contribution of various signaling pathways in human tissue development and homeostasis. Established organoids can also be used to study host-microbe interaction and even responds to environmental and food processing-generated toxins. When incorporated with disease related information by biochemical and genetic manipulations, human disease models can be established and used for identification of driver mutations and key pathophysiology pathways in disease conditions. Furthermore, organoids carrying specific disease information of a patient can be grown in large scale for preclinical drug screenings for treatment with high efficacy and minimal side-effect that being tailored to the patient’s requirement (Fig. 2A). Importantly, biobanks are being established collecting human organoids that can potentially offer organoids of human populations, which can be used for preclinical efficacy and toxicity tests of candidate drugs, screen for diagnostic and prognostic factors, and study of genotype-phenotype causality in conjunction with various genetic studies (e.g. genome-wide association studies) (Fig. 2B). Moreover, it is also clear that organoid technologies offer a potential large source of donor tissues for transplantation use.

Complemented with advances in bioengineering approaches, it is possible to build up organoids from different tissues together or even a functional gastrointestinal system using microfludic devices termed “organs-on-a-chip” (Fig. 2C). Although still early in their development, several organ-on-a-chip assay formats have already been evaluated. For example, a four-organ-chip system was established that included human liver, skin, intestine and kidney (Maschmeyer et al., 2015). It is foreseen that generation of such complex, stem-cell-based, multi-organ *in vitro* system would allow us to model much more complicated physiological processes, such as human nutriology (Ben-Zvi and Melton, 2015).

Genome editing technology emerges as an extremely powerful tool that can greatly advance organoid-based research and therapies. With various genome editing tools, genetic variants, epigenetic alterations, or even changes in chromatin structure can be efficiently corrected from patient cells or introduced into wild-type cells. A variety of human disease models can therefore be established for pathophysiology study and treatment development. Besides, different high-throughput genome editing platforms are offering remarkable resources that can be adapted to screen for potential therapeutic targets in combination with disease models. It is also possible that organoids can be edited to possess extra functions that facilitate the *in vitro* organoid formation and maturation. For instance, certain support cells can be edited to enhance niche function or to cooperate better with biomimetic scaffolds. Perhaps the most exciting application lies in organoid-based therapeutics in the future. Organoids derived from patients with hereditary diseases can be “cured” with genome editing to correct the disease-causing mutation(s), and functional organoids can then be transplanted back into patients to improve tissue functionality. Altogether, although challenges exist that the current organoids system still need substantial development, this system have already found utility in many basic biological and therapeutic fields to advance new knowledge and to advance us closer to therapeutics for diseases that previously appeared untouchable.
